# MDR-TB patients in KwaZulu-Natal, South Africa: Cost-effectiveness of 5 models of care

**DOI:** 10.1371/journal.pone.0196003

**Published:** 2018-04-18

**Authors:** Marian Loveday, Kristina Wallengren, Tarylee Reddy, Donela Besada, James C. M. Brust, Anna Voce, Harsha Desai, Jacqueline Ngozo, Zanele Radebe, Iqbal Master, Nesri Padayatchi, Emmanuelle Daviaud

**Affiliations:** 1 Health Systems Research Unit, South African Medical Research Council, Cape Town, South Africa; 2 Centre for the AIDS Programme of Research in South Africa (CAPRISA), SA Medical Research Council Extramural TB Pathogenesis Research Unit, University of KwaZulu-Natal, Durban, South Africa; 3 TB and HIV Investigative Network (Think), Durban, South Africa; 4 Biostatistics Unit, South African Medical Research Council, Cape Town, South Africa; 5 Montefiore Medical Center & Albert Einstein College of Medicine, Bronx, New York, United states of America; 6 Discipline of Public Health Medicine, University of KwaZulu-Natal, Durban, South Africa; 7 Deloitte Consulting, South Africa, Johannesburg, South Africa; 8 KwaZulu-Natal Department of Health, Pietermaritzburg, South Africa; 9 King Dinuzulu Hospital, KwaZulu-Natal Department of Health, Durban, South Africa; Agencia de Salut Publica de Barcelona, SPAIN

## Abstract

**Background:**

South Africa has a high burden of MDR-TB, and to provide accessible treatment the government has introduced different models of care. We report the most cost-effective model after comparing cost per patient successfully treated across 5 models of care: centralized hospital, district hospitals (2), and community-based care through clinics or mobile injection teams.

**Methods:**

In an observational study five cohorts were followed prospectively. The cost analysis adopted a provider perspective and economic cost per patient successfully treated was calculated based on country protocols and length of treatment per patient per model of care. Logistic regression was used to calculate propensity score weights, to compare pairs of treatment groups, whilst adjusting for baseline imbalances between groups. Propensity score weighted costs and treatment success rates were used in the ICER analysis. Sensitivity analysis focused on varying treatment success and length of hospitalization within each model.

**Results:**

In 1,038 MDR-TB patients 75% were HIV-infected and 56% were successfully treated. The cost per successfully treated patient was 3 to 4.5 times lower in the community-based models with no hospitalization. Overall, the Mobile model was the most cost-effective.

**Conclusion:**

Reducing the length of hospitalization and following community-based models of care improves the affordability of MDR-TB treatment without compromising its effectiveness.

## Introduction

Multidrug-resistant tuberculosis (MDR-TB), defined as TB resistant to isoniazid and rifampicin, threatens global TB control [1]. Although MDR-TB represents only 7% of incident TB in South Africa, high drug prices, lengthy treatment and hospitalization lead to exorbitant costs, and in 2014, approximately 65% of the National Tuberculosis Program budget was spent on MDR-TB control [1–3].

A number of global studies, including two systematic reviews, have reported higher costs associated with managing MDR-TB patients in hospital [2, 4–7]. Although a number of studies recommend community-based models of treatment and limiting hospitalization, few compare models of care in large cohorts without having to rely on some hypothetical implementation.

The province of KwaZulu-Natal has amongst the highest prevalence of patients with MDR-TB in South Africa [8]. Until 2008, local management of MDR-TB required hospitalization in a centralized specialized hospital, but the rising caseload rendered this model of care impractical. In 2008, new models of care were implemented in certain areas of the province (Table 1): decentralized care (rural district hospitals with non-specialist doctors providing hospitalization and care), and community-based care (patients were not hospitalized, but treated in their homes by a mobile injection team or at the nearest clinic). In other areas, the existing centralized model of care (specialized referral center providing hospitalization and subsequent care), remained in place. Decentralized and community-based models of care were introduced to increase accessibility of MDR-TB services, reduce the duration of hospitalization and enable all patients to commence treatment immediately without waiting for a hospital bed. Monitoring was poor, and therefore, interpretation and implementation of the new models of care varied [9]. Health care workers anxious about MDR-TB transmission in the community hospitalized patients for longer than necessary [10], and, despite of a list of criteria detailing which patients could receive community-based treatment, hospitalized most patients (S1 Table) [11].

**Table 1:** Models of care for MDR-TB patients in KwaZulu-Natal 2009–2012.

Models of Care	Level of care	Length of hospitalization	MDR-TB*	Clinic Visits	Mobile Visits
			OPD^†^ Visits		
Centralized hospital	Specialized Hospital	Initial hospitalization for all patients	Monthly MDR-TB OPD visits at centralized hospital after discharge as an inpatient	If patient discharged during intensive phase received injectable at local clinic.	Not applicable
Decentralized 2	District Hospital	Hospitalization for all patients for whole injectable phase	After discharge monthly OPD visits	Not applicable	Not applicable
Decentralized 1	District Hospital	Initial hospitalization for all patients	After discharge monthly OPD visits	If patient discharged during intensive phase received injectable from local clinic or a mobile.	Not applicable
Community-based	Clinic	No hospitalization for any patient	Monthly at decentralized Hospital	During intensive phase received injectable from local clinic.	Not applicable
Community-based	Mobile	No hospitalization for any patient	Monthly at decentralized Hospital	Not applicable	During intensive phase received injectable from mobile.

* MDR-TB: Multidrug-resistant TB, TB resistant to isoniazid and rifampicin;

† OPD: Outpatient department

In a previous study we evaluated the effectiveness of the new program in decentralized hospitals, reporting that MDR-TB patients were more likely to have a successful treatment outcome if they received decentralized care, compared to traditional care at a central specialized hospital (adjusted OR = 1·43, p = 0·01) [10]. The cost-effectiveness of the various models, however, was still unknown. Given the size of the MDR-TB epidemic and the cost of treatment, determining cost-effectiveness is critical for policy makers and TB program managers. In this study, we defined cost-effectiveness as provider costs per successfully treated patient. We then addressed the following question: which model of care is most cost-effective?

## Methods

### Study population: Patients and health facilities

In our prospective cohort study, all MDR-TB patients ≥ 18 years who started treatment between July 2008 and July 2010 were enrolled. Patients were excluded if they had additional resistance to a fluoroquinolone or a second-line injectable agent (i.e., pre-extensively drug-resistant TB [pre-XDR TB]) or both (XDR TB). All patients who lived within the catchment area of the decentralized site were enrolled at that site if they met the study criteria. At the centralized hospital, all patients who met the study criteria were enrolled, unless they came from the catchment areas of a decentralized site.

MDR-TB patients were treated in one of the 5 models of care available at the time (Table 1). Most patients at the centralized and decentralized sites were initially hospitalized. However, at all three hospitals there were a few patients who met the criteria for community-based treatment (S1 Table) and were not hospitalized, receiving all their treatment from their closest clinic or a mobile injection team. We grouped all patients from the 3 hospitals who were not hospitalized into a community-based model (clinic or mobile). The remaining patients were assigned to the hospital to which they initially presented, were hospitalized and subsequently managed. The new models of care were geographically positioned throughout the province, with a strategic focus on areas with the highest incidence of MDR-TB [12]. Infrastructure and the socio-economic profile of the populations in these areas was similar [13] (S1 Appendix). Four decentralized sites started treating patients with MDR-TB in 2008. As their performance varied considerably [10], for our cost-effectiveness study, we included only the best and worst performing of these hospitals to account for the range of variability.

### Data collection

We collected patient data from medical notes and the MDR-TB treatment register. For each patient, we collected duration of treatment and hospitalization, length of intensive and continuation phases, as well as HIV status and receipt of antiretroviral therapy (ART).

Using a provider perspective, we collected costs to the health service only, excluding household costs. For the hospitals, we collected recurrent costs data, broken down by category (clinical staff, drugs, laboratory tests, catering and laundry) and indirect service costs (non-clinical staff and overheads). From the MDR-TB ward and MDR-TB outpatient department (OPD), we collected the number of Full-Time Equivalents (FTEs) per category of staff. We collected similar financial information for the clinics and mobile injection teams. For the mobile teams we included the capital cost of the vehicles. Financial data were extracted from the KwaZulu-Natal provincial department of health accounts (2012).

Diagnostic and treatments costs included baseline diagnostic costs, medication required during the 6-month intensive phase and 18-month continuation phase, ART for HIV co-infected patients and the costs of routine monitoring at each monthly check-up. Unit costs of laboratory tests were extracted from the National Health Laboratory Service (NHLS), state pricing list (2012–13). Costs of chest x-rays and audiograms were obtained from the hospitals’ financial data. Drug costs were extracted from the KwaZulu-Natal central medical depot pricing list (2012–13). Inpatient day (IPD) costs were calculated primarily from three categories of expenditure: clinical staff, direct service costs (laundry, catering), and indirect service costs. OPD costs were derived from direct personnel costs and indirect service costs.

Activity data by facility were provided by the KwaZulu-Natal provincial department. Data on number and type of contacts made by mobile teams were extracted from their registers.

### Study outcomes

Treatment outcomes of patients were determined at the end of treatment, according to definitions developed by the WHO (S2 Table) [14, 15]. Treatment success was defined as the proportion of patients who were cured or completed treatment.

The primary outcomes of this study were cost-effectiveness and incremental cost-effectiveness ratios (ICER). We defined cost-effectiveness as provider costs per successfully treated patient compared to a no treatment option and present this cost for each model of care. ICERs were used to compare the cost-effectiveness of the different models of treatment [16].

### Data analysis

We initially had 1269 patients in our study, but excluded 231 patients at the Centralized hospital who had missing data on hospital duration. The remaining 1038 patients were included in the cost-effectiveness analysis.

We calculated the economic costs for each patient using several steps. Firstly, we determined the type and number of contacts with the health service: numbers of IPDs, OPD visits, number of days in the intensive phase to determine the number of injections administered and number of days in the continuation phase to determine the number of OPD visits for monthly monitoring.

We calculated the unit cost per type of health service contact: IPD, OPD visit, injection in clinics or at home with mobiles, from which we quantified the cost for each patient. The cost per patient for drugs, diagnostics and monitoring tests (laboratory, audiology and chest x-rays) was calculated according to standard treatment protocols. We added, where relevant, the cost of HIV related services: HIV testing, CD4 cell count and viral load tests as well as ART associated costs. Cost of pregnancy tests for all women aged 15 to 49 were included.

For the hospitals, we obtained clinical staff cost per IPD and OPD visit, by applying the mid-point salary package to the Full-Time Equivalents (FTEs) per category of staff [17]. This was then divided by the total number of IPDs or OPD visits for the year. Catering, laundry and indirect costs were calculated using each hospital’s average per IPD and OPD visit [18] (S3 Table).

We calculated recurrent cost per injection at clinics using the average cost per consultation at the clinic. For mobiles, the cost of home injections was the sum of the annualized mobile capital and running costs, the cost of the nurse running each mobile, with an additional 10% overheads for planning and management. These costs were apportioned by applying the proportion of MDR-TB injection visits. Capital costs were annualised over 5 years using a 3% discount rate. For each patient the cost of diagnosis and treatment was calculated as follows:

Cost of Tests and Drugs + (Cost clinical staff per IPD + Other costs per IPD) * number of IPDs + (Cost clinical staff per OPD + Other costs per OPD) * number of OPD visits + Cost per clinic injection * number of clinics injections OR Cost per mobile injection * number of mobiles injections.

Data collected in 2012 ZAR (the last year of our study) were inflated to 2014 using the South African medical consumer price index of 6·3 for 2012 and 6·4 for 2013 [19]. This was converted to US dollars using the 2014 average annual exchange rate of USD1 = South African rand (ZAR) 10·44 [20].

Baseline characteristics and treatment outcomes were described using simple frequencies. Where appropriate Chi-square and Fisher’s exact test, were used to test the relationship between categorical characteristics and model of care. All baseline factors indicative of an imbalance in treatment groups were further analysed for their individual effects on cost and effectiveness (treatment success), respectively.

The propensity score is a balancing tool to reduce differences in the distribution of baseline variables between treatment groups [21]. Due to the limited sample size in the mobile and clinic models, propensity score weighting presented a more feasible approach than propensity score matching which may result in the exclusion of patients. We created, using the variables previous TB treatment, HIV and ART status, baseline weight and positive smear microscopy in logistic regression, separate propensity scores to compare the following pairs of treatment groups: Centralised vs Decentralised 2, Clinic vs Decentralised 2, Mobile vs Clinic, Decentralised 1 vs Mobile. These four comparisons were ordered, according to effectiveness, from lowest to highest for the purpose of the ICER analysis. Using the overidentification test, the balance of covariates between treatment models was assessed and, having adjusted for the propensity score weights, the standardized differences of covariates were compared. All individuals with missing data for any of the covariates were excluded for this weighting exercise (S2 Appendix).

In the ICER analysis only propensity weighted costs and treatment success rates are presented. Having ordered the models of care by success rates (lowest to highest), each model was compared to the previous model to determine which model was most cost-effective, i.e. had the lowest provider cost per successfully treated patient. Two sensitivity analyses were conducted to determine at which success rate and what number of days of hospitalization the hospitalized models would become as cost-effective as the most cost-effective model.

### Ethics approval

The study protocol was approved by the University of KwaZulu-Natal Biomedical Research Ethics Committee and the KwaZulu-Natal Department of Health. Informed consent was waived by the ethics committee, as all data had been previously collected during routine medical care and did not pose any additional risks to patients.

## Results

### Patient characteristics and treatment outcomes

Of the 1038 MDR-TB patients studied 52% were female and the median age was 35 years [IQR 27–43] (Table 2). HIV co-infection rates varied across the different models of care, from 84% at Decentralized 2 to 62% in the Mobile model (p = 0.002). The proportion of HIV-infected patients receiving ART across the modes of care varied, with 77% of patients at Decentralized 2 receiving ART compared to 100% in the Clinic and Mobile models (p<0.001). (Receipt of ART was a criterion for admission to the community-based models for patients co-infected with HIV). In our study cohort 114/748 (15%) of HIV-infected patients were not on ART (Table 3). There was variation in other baseline variables across the models of care, but only the differences in pre-treatment weight (p<0.001) and previous episodes of TB (p<0.001) were significant. As HIV and ART status had a significant effect on treatment outcomes, we stratified treatment outcomes by HIV and ART status (Table 3). As expected, patients co-infected with HIV not on ART had poorer treatment outcomes than those who were on ART.

**Table 2:** Baseline demographic and clinical characteristics of MDR-TB patients (N = 1038).

Patient characteristics	Centralized hospital	Decentralized models		Community-based models		
		1	2	Clinic	Mobile	p-value
	N = 582	N = 125	N = 261	N = 25	N = 45	
Female	299 (51)	68 (54)	136 (52)	13 (52)	23 (51)	0.88
Median age (years, IQR)	34 (27–41)	36 (28–42)	36 (29–44)	34 (29–42)	33 (28–42)	0.3270
Median weight (kg, IQR)	53 (46–60)	49 (43–56)	52 (44–59)	48 (41–52)	52 (47–59)	0.0002
Previous TB	558 (96)	87 (70)	107 (41)	18 (72)	26/45 (58)	<0.001
HIV-infected, n/total tested*	411/564 (73)	96/124 (77)	197/235 (84)	16/24 (67)	28/45 (62)	0.002
On ART, n/known ART status^†^	331/404 (82)	92/95 (97)	129/167 (77)	16/16 (100)	28/28 (100)	<0.001
Smear positive at diagnosis	406 (52)	80 (64)	195 (75)	16(64)	25(56)	<0.001
Resistant to ≥ 3 drugs at baseline	470 (58)	72(58)	138(53)	15(60)	24 (53)	0.677

Data are number (%) unless otherwise stated.

* Unknown HIV status documented in: 18 patients in the centralized hospital; 1 patient in Decentralized 1; 26 patients in Decentralized 2; l patient in the Clinic and 0 patients in the Mobile models.

^†^ Unknown ART status documented in: 7 patients in the centralized site, 1 patient at Decentralized 1 and 30 patients Decentralized 2.

**Table 3:** Treatment outcomes of patients with MDR-TB in KwaZulu-Natal, South Africa (N = 1038).

	Cured	Completed treatment	Treatment success^†^	Died	Failed	Defaulted	Transferred out
**Centralized hospital (n = 582)**							
Total	198 (34%)	117 (20%)	315 (54%)	101 (17%)	19 (3%)	145 (25%)	2
HIV-negative	55 (36%)	34 (22%)	89 (58%)	20 (13%)	3 (2%)	41 (27%)	0
HIV-positive + ART	116 (35%)	71 (22%)	187 (57%)	57 (17%)	12 (4%)	73 (22%)	2 (0.6%)
HIV-positive no ART	17 (23%)	10 (14%)	27 (37%)	16 (22%)	4 (5%)	26 (36%)	0
Unknown HIV status	7 (39%)	2 (11%)	9 (50%)	6 (33%)	0	3 (17%)	0
HIV-positive, unknown ART status	3 (43%)	0	3 (43%)	2 (29%)	0	2 (29%)	0
**Decentralized 1 (n = 125)**							
Total	78 (62%)	12 (10%)	90 (72%)	17 (14%)	7 (6%)	9 (7%)	2 (2%)
HIV-negative	17 (61%)	3 (11%)	20 (72%)	2 (7%)	2 (7%)	3 (11%)	1 (4%)
HIV-positive + ART	58 (63%)	9 (10%)	67 (73%)	14 (15%)	5 (5%)	5 (5%)	1 (1%)
HIV-positive no ART	2 (67%)	0	2 (67%)	0	0	1 (33%)	0
Unknown HIV status	1 (100%)	0	1 (100%)	0	0	0	0
HIV-positive, unknown ART status	0	0	0	1 (100%)	0	0	0
**Decentralized 2 (n = 261)**							
Total	120 (46%)	15 (6%)	135 (52%)	69 (26%)	19 (7%)	28 (11%)	10 (4%)
HIV-negative	18 (47%)	2 (5%)	20 (52%)	6 (16%)	3 (8%)	8 (21%)	1 (3%)
HIV-positive + ART	72 (56%)	8 (6%)	80 (62%)	24 (19%)	10 (8%)	12 (9%)	3 (2%)
HIV-positive no ART	16 (42%)	2 (5%)	18 (47%)	11 (29%)	2 (5%)	4 (11%)	3 (8%)
Unknown HIV status	4 (15%)	2 (8%)	6 (23%)	13 (50%)	3 (12%)	2 (8%)	2 (8%)
HIV-positive, unknown ART status	10 (33%)	1 (3%)	11 (37%)	15 (50%)	1 (3%)	2 (7%)	1 (3%)
**Clinic (n = 25)**							
Total	12 (48%)	3 (12%)	15 (60%)	4 (16%)	2 (8%)	4 (16%)	0
HIV-negative	0	1 (12%)	1 (12%)	4 (50%)^¥^	2 (25%)	1 (4%)	0
HIV-positive + ART	12 (75%)	2 (12%)	14 (87%)	0	0	2 (8%)	0
HIV-positive no ART	0	0	0	0	0	0	0
Unknown HIV status	0	0	0	0	0	1 (100%)	0
HIV-positive, unknown ART status	0	0	0	0	0	0	0
**Mobile (n = 45)**							
Total	30 (67%)	0	30 (67%)	4 (9%)	3 (7%)	5 (11%)	3
HIV-negative	6 (35%)	0	6 (35%)	4 (23%)	3 (18%)	3 (18%)	1 (6%)
HIV-positive + ART	24 (86%)	0	24 (86%)	0	0	2 (7%)	2 (7%)
HIV-positive no ART	0	0	0	0	0	0	0
Unknown HIV status	0	0	0	0	0	0	0
HIV-positive, unknown ART status	0	0	0	0	0	0	0

^†^ Treatment success: Sum of the patients cured and completed treatment.

^¥^ Two patients died due to trauma, deaths not related to TB.

### Treatment details and costing

Duration of treatment, length of intensive phase, and length of hospitalization varied between patients, affecting the number of injections administered to outpatients and the number of OPD visits (Table 4). In Table 5, the mean cost per patient per type of activity and per model are shown.

**Table 4:** Days of treatment and numbers of attendances of MDR-TB patients (N = 1038).

	Centralized	Decentralized models		Community-based models	
	hospital	1	2	Clinic	Mobile
Duration of MDR-TB* treatment (days)	482/595	583/719	499/664	474/687	575/693
Intensive phase					
Duration (days)	187/196	177/195	167/182	164/189	179/192
Inpatient days	130/136	79/70	158/174	0	0
Injections administered in the community (days)	57/60	98/125	9/8	164/189	179/192
Number hospital OPD^†^ visits	13/3	4/4	1/1	6.1/7	6.5/7
Number clinic injections	42/36	36/39	7/0	117/135	0
Number mobile injections	0	35/39	0	0	127/137
Continuation phase					
Duration (days)	311/392	405/504	342/484	309/462	396/495
Number OPD Visits	9/12	14/17	11/16	10/15	13/16

Data are mean/median

* MDR-TB: Multidrug-resistant TB, TB resistant to isoniazid and rifampicin;

^†^ Outpatient department

**Table 5:** Cost per MDR-TB patient treated for each care model in 2014 US dollars (USD)* (N = 1038).

	Centralized	Decentralized models		Community-based models	
	hospital	1	2	Clinic	Mobile
IPD Cost (USD)	25,282	12,631	24,130	0	0
OPD Cost (USD)	1,071	1,879	1,086	1,727	2,071
Clinic/Mobile (USD)	636	1,059	116	1,758	3,447
Tests^†^ (USD)	1,256	1,545	1,097	1,293	1,534
Drugs (USD)	1,940	2,371	2,013	1,961	2,342
Total cost (USD)	30,185	19,484	28,246	6,739	9,394

*Mean costs;

^†^Tests: diagnostic and monitoring tests—laboratory, audiology and chest x-rays

The total cost per patient treated varied significantly across the models of care (Decentralized hospital 30,185USD, Decentralized 2 28, 246USD, Decentralized 1 19, 484USD, Mobile 9,394USD and Clinic 6,739USD; p<0.0001), with the average cost per patient 3 to 4.5 times lower in the community-based models of care. Inpatient care (excluding drugs and labs) was the main cost driver in the hospital-based models, accounting for 85% of cost per patient treated at Decentralized 2, 84% at the Centralized hospital and 65% at Decentralized 1. In contrast, in the community-based models of care, tests and drugs accounted for 41% and 48% of the costs in Mobile and Clinic care respectively. The length of hospitalization accounted for the difference in the cost per patient treated at the two decentralised models. At Decentralized 2 the mean number of inpatient days was twice that of Decentralized 1 (158 vs 79 days) (Table 4), with an IPD cost per patient almost twice that of Decentralized 1 (USD24,130 vs USD12,631) (Table 5).

Having ranked the models of care from lowest to highest treatment success, the following comparisons were used for the ICER analysis: Centralized vs Decentralized 2, Decentralized 2 versus the Clinic model, the Clinic versus the Mobile model and finally the Mobile versus Decentralized 1. After applying propensity score weights for each of the aforementioned comparisons separately, previous TB, HIV and ART status, baseline weight and positive smear microscopy were similar between the models of care. (The standardized differences between covariates before and after weighting as well as the p-values for covariate balance are presented in S2 Appendix.)

Propensity score weighted costs and treatment success rates are presented in Table 6. The Centralized model of care was the least cost-effective owing to the lowest success rate and the highest cost per patient. This was followed by Decentralized 2. The community-based models (Clinic and Mobile) were more cost-effective than models which included hospitalisation. Although the Mobile model was more costly than the Clinic model, it was 8% more effective, so that overall, the Mobile model was the most cost-effective model. Although Decentralized 1 had a 1% higher success rate than the Mobile model it was substantially more expensive than the Mobile.

**Table 6:** Cost-effectiveness of the 5 models of care in 2014 US dollars (USD) using propensity score weighted costs and treatment success rate*.

Model of care	Success rate	Cost per patient (USD)	Average cost per success (USD)	ICER (USD)	Interpretation
Centralized	59%	30575	51822	-179	Decentralized 2 is more cost-effective than the Centralized model.
Decentralized 2	63%	29858	47394		
Decentralized 2	58%	29200	50345	-2738	The Clinic model is more cost-effective than Decentralized 2.
Clinic	66%	7297	11056		
Clinic	60%	6626	13943	402	The Mobile model is more effective but more costly than the Clinic model. The 8% difference in treatment success justifies the increased cost of the mobile model.
Mobile	68%	9841	11043		
Mobile	72%	9957	13829	9687	Decentralized 1 is more costy than the Mobile model. The 1% difference in treatment success does not justify the increased cost of Decentralized 1.
Decentralized 1	73%	19644	26910		

Final interpretation: From this analysis, the Mobile model is overall the most cost-effective model.

* Note: The four comparisons (Decentralized vs Decentralized 2, Decentralized 2 vs Clinic, Clinic vs Mobile and Mobile vs Decentralized 1) were considered separately in propensity score analysis to match patients on their demographic and health baseline factors

Having identified treatment success and length of hospitalization as the two variables with the greatest impact on cost per patient successfully treated, we adjusted these two variables to assess their impact on cost-effectiveness of the models. Even if treatment success was increased to 100% in the Centralized and Decentralized models, they remained significantly more expensive than the community-based models. If the days of hospitalization at the Centralized hospital was reduced to 2, the cost per successfully patient treated was still higher than that of the community-based models. For Decentralized 1 and 2, the days of hospitalization had to be reduced from 79 to 5 days and 158 to 6 days respectively for the cost for each successfully treated patient to be equivalent to that of the community-based models.

## Discussion

Our large study, involving 1,038 MDR-TB patients, shows that community-based care is more cost-effective than care in either a decentralized or centralized setting as evidenced by the lower cost per patient successfully treated in the community-based models of care. Overall, the Mobile model was the most cost-effective model. Our findings support the recent WHO recommendation, together with that of others, for ambulatory care as the preferable model of care for patients with MDR-TB [22–24].

Our study compared the cost-effectiveness of 5 different models of care in South Africa based on actual implementation and individual patient data. Four other South African studies have attempted to cost models of care, but relied on some estimated data, hypothetical implementation or data from one program only [6, 7, 25, 26]. The costs we recorded are higher than those reported in these studies, probably due to longer treatment durations and higher HIV co-infection rates.

In our study, in the models of care in which patients were hospitalized (centralized and decentralized), hospitalization was a major cost driver, accounting for 65–85% of treatment costs. Even after controlling for duration of treatment, the cost per patient was significantly lower in the community-based models than the models which included hospitalization. In our setting, however, there will always be patients who require hospitalization. A number of global studies support these findings. In a systematic review inpatient care was estimated to cost 1.6 times more than community-based care [27]. Fitzpatrick et al report that community-based care incurred lower costs than inpatient care and was more cost-effective [2]. They recommend community-based care unless there is strong evidence for hospitalization. A study in Estonia and Russia found that hospitalization costs accounted for 67–82% of their total treatment costs [5], a finding very similar to our study.

In the community-based models of care in our study—in which patients were not hospitalized—tests and drugs were a major cost driver, accounting for 41% of the cost of mobile services and 48% of the cost of clinic services. The costs we report for tests and drugs are similar to those reported by one of the South African studies [25]. We report higher costs for tests and drugs than Sinanovic et al, which is probably due to longer treatment durations and higher HIV co-infection rates [26].

Patient enrolment in the study started in 2008 and limited access to ART at that time accounts for the 15% of HIV-infected patients who were not on ART. ART is now more easily accessible as eligibility criteria have changed and nurses trained to initiate ART. Furthermore, with the introduction of the new test and treat approach in South Africa, there will soon be very few HIV-positive patients not on ART. In patients co-infected with HIV, ART is a significant determinant of treatment success [28, 29], and in our study 67 (59%) of HIV-positive patients not on ART has an unsuccessful treatment outcome.

Surprisingly, the community-based models of treatment were not more effective for HIV-negative patients. This, however, may be a consequence of the small number of HIV-negative patients in our cohort and that 2 of the 4 deaths (out of 8 total HIV-negative patients in the clinic model), were unrelated to MDR-TB (Table 3).

The differences between the two decentralised models highlight that alternate models of care are not always more effective or more cost-effective. Treatment success was lower at Decentralized 2 (Table 3). And, although decentralization aimed to reduce the length of hospitalization, Decentralized 2 reported long periods of hospitalization (a mean of 158 days) as clinicians, unconfident about managing MDR-TB and anxious about MDR-TB transmission in the community, hospitalised patients for longer than necessary or stipulated in the guidelines. These findings highlight the need for regular monitoring and support during service expansion to ensure staff understand new programs and implementation is according to guidelines. Numerous studies have reported the difficulties in introducing and expanding new diagnostics, algorithms or models of care [30, 31].

Community-based treatment together with decreasing the length of hospitalization reduces provider costs of MDR-TB services. However, as we did not capture household costs, we were unable to determine which model of care was most cost-effective to patients and society overall. Although the diagnosis and treatment of MDR-TB in South Africa is free, patients incur substantial costs accessing health services, with the poorest patients incurring the highest costs [32, 33]. In some instances, when patients can continue with their household duties and return to work when they respond to treatment, community-based treatment will reduce household costs. In other instances, however, significant household costs may be incurred accessing the clinic daily or nursing an ill patient at home. As patients with TB are poorer than the average South African [34] and social protection against the cost of illness is a key objective of the post-2015 Global TB strategy [35], to optimize the chance of treatment success [36] and reduce catastrophic costs, the mechanism for delivery of all MDR-TB services must minimise productivity loss and provide timely social protection. In promoting community-based models of treatment, education on infection control at a household level is essential to minimize possible transmission, the infection of a household member and additional household-level costs.

As our study reports findings from a large study cohort, of a programme implemented and funded entirely by the Department of Health, at sites with heterogeneous treatment success, we believe this increases the generalizability of our findings to other resource-limited settings.

This operational study evaluated an intervention implemented by the public sector, and we had limited control over the design, scope and quality of implementation. Many patients were excluded from our analysis due to missing data. The generalisability of our findings is limited by the small number of patients treated in the community-based models and that there were no HIV-infected patients not on ART in the community-based models. Additional adequately powered studies are needed to better inform criteria for allocation to ambulatory care and to determine which models of care are most effective in differing community contexts, as are those investigating household costs related to MDR-TB.

We conclude that even in resource-limited settings and in the presence of HIV co-infection, community-based care is more cost-effective than care in either a centralized or decentralized hospital setting for patients who do not require hospitalization. As the global number of MDR-TB patients continues to increase, our findings support the WHO call for ambulatory care.

Ambulatory care reduces the provider costs of MDR-TB treatment and possibly household costs too. Recent advances in technology, including short course regimens, new and repurposed drugs and mobile phones have the potential to reduce provider and household costs further. To assess the impact of these new technologies on provider and household costs, additional cost effectiveness studies should be performed as these interventions are implemented. However, providing effective MDR-TB care requires a complex health system response, the complexity of which will increase as new drugs and diagnostic tools emerge. Recognising that different models of care are required to provide universal access to MDR-TB treatment, responsible oversight and vigilance by National TB programs, together with appropriate investment in health systems and staff is necessary to ensure all MDR-TB services are effective.

## Supporting information

S1 Table Criteria for home-based treatment of MDR-TB patients. (DOCX)

S2 Table Treatment outcome definitions. (DOCX)

S3 Table Unit cost per type of activity per site. (DOCX)

S1 Appendix Comparison of populations included in this study. (DOCX)

S2 Appendix Additional methodology regarding the propensity analysis. (DOCX)
