# Supplementary material for: MDR-TB patients in KwaZulu-Natal, South Africa: Cost-effectiveness of 5 models of care
Source: PLoS One. 2018 Apr 18;13(4):e0196003. doi: 10.1371/journal.pone.0196003 (PMC5906004; doi:10.1371/journal.pone.0196003)
Supplement: S1 Table — (DOCX) [file pone.0196003.s001.docx]

| **S1 Table: Criteria for home-based treatment of MDR-TB patients** | |
| --- | --- |
| **1.** | Patient ambulant; |
| **2.** | Patient a low grade transmission risk (smear negative); |
| **3.** | Patient had access MDR-TB initiation site monthly; |
| **4.** | Patient accessible to mobile MDR-TB team; |
| **5.** | Patient had stable accommodation; |
| **6.** | Patient had adequate nutritional support; |
| **7.** | Household member present - for treatment support in the home; |
| **8.** | Patient had a good reason for not wanting to be hospitalized. |
