# Supplementary material for: MDR-TB patients in KwaZulu-Natal, South Africa: Cost-effectiveness of 5 models of care
Source: PLoS One. 2018 Apr 18;13(4):e0196003. doi: 10.1371/journal.pone.0196003 (PMC5906004; doi:10.1371/journal.pone.0196003)
Supplement: S2 Table — (DOCX) [file pone.0196003.s002.docx]

| **S2 Table: Treatment outcome definitions*** | |
| --- | --- |
| **Treatment outcome** | **Definitions** |
| Cure | Completion of treatment and >5 consecutive negative culture results in the final 12 months of treatment. |
| Treatment completion | Completion of therapy but without bacteriologic documentation of cure. |
| Treatment failure | More than one positive culture in the final 12 months of therapy, or if any one of the final three cultures was positive, or if more than one drug in the treatment regimen was replaced, or if treatment was terminated due to adverse events or no clinical improvement. |
| Default | Interruption in treatment for > 2 consecutive months for any reason. |
| Death | All-cause mortality during MDR TB treatment. |
| Transferred out | Patients transferred to another reporting and recording unit a year after study-enrolment whose treatment outcome is unknown. |
| Treatment success | Treatment success is the percentage of patients in whom the treatment outcome was either cured or completed. |
| Unsuccessful treatment | Percentage of patients in whom the treatment outcome was died, defaulted, or failed treatment. |

*Treatment outcome definitions used are WHO definitions for the management of MDR TB.^4,5^
