# Supplementary material for: MDR-TB patients in KwaZulu-Natal, South Africa: Cost-effectiveness of 5 models of care
Source: PLoS One. 2018 Apr 18;13(4):e0196003. doi: 10.1371/journal.pone.0196003 (PMC5906004; doi:10.1371/journal.pone.0196003)
Supplement: S3 Table — (DOCX) [file pone.0196003.s003.docx]

| **S3 Table: Unit cost per type of activity per site** (all costs in US dollars) | | | | | |
| --- | --- | --- | --- | --- | --- |
|  | **Centralized hospital** | **Decentralized models** | | **Community-based care** | |
|  |  | **1** | **2** | **Clinic** | **Mobile** |
| **Cost per inpatient day (IPD)** | | | | | |
| Clinical staff costs^†^ | 74 | 89 | 75 |  |  |
| Catering and laundry^‡^ | 6 | 5 | 7 |  |  |
| Indirect costs^‡^ | 114 | 66 | 71 |  |  |
| Total | 194 | 161 | 153 |  |  |
| **Cost per outpatient visit (OPD)** | | | | | |
| Clinical staff costs^†^ | 49 | 84 | 47 |  |  |
| Indirect costs^‡^ | 38 | 22 | 34 |  |  |
| Total | 87 | 106 | 81 |  |  |
| **Cost/Injection** | | | | | |
| Clinical staff costs |  |  |  | 21 | 4 |
| Mobile |  |  |  | 0 | 21 |
| Indirect costs^‡^ |  |  |  | 3 | 2 |
| Total |  |  |  | 15 | 27 |

^†^Department of Public Service and Administration. Salary Scales – Annexure C: Nursing Staff and

Annexure I. In: National Department of Public Service and Administration, ed. Pretoria, South Africa 2012.

^‡^KwaZulu-Natal Department of Health Financial Reports. Pietermaritzburg, KwaZulu-Natal 2012.
