# Supplementary material for: MDR-TB patients in KwaZulu-Natal, South Africa: Cost-effectiveness of 5 models of care
Source: PLoS One. 2018 Apr 18;13(4):e0196003. doi: 10.1371/journal.pone.0196003 (PMC5906004; doi:10.1371/journal.pone.0196003)
Supplement: S1 Appendix — (DOCX) [file pone.0196003.s004.docx]

**S1 Appendix: Comparison of populations included in this study**

South Africa has 9 provinces which are divided into 52 districts. We have a district health system and primary health care services are available for the district population within the district and managed within the district. Our study was based in one province - KwaZulu-Natal. At the time of our study, 4 of KwaZulu-Natal province’s 11 districts had a decentralised MDR-TB unit and managed all patients diagnosed with MDR-TB in their district/catchment area. The centralised hospital managed patients with MDR-TB from the other 7 districts.

To compare the populations served by the different models of care, to determine if at baseline the populations were comparable, we used a Deprivation Index as a composite index of deprivation together with a number of more specific health status indicators.

The Deprivation Index (DI) was based on community level data obtained during the 3 years prior to the commencement of our study.^1^ “The DI is a measure of relative deprivation of populations across districts within South Africa, and was derived from a set of demographic and socio-economic variables obtained from the 2007 Community Survey and the 2005 and 2006 General Household Surveys. The deprivation index was generated with health care resource allocation in mind, therefore the variables used were those that could also serve as indicators of need for health care resources.”^1^ A number of indicators of deprivation were reduced into a composite index of deprivation which reflected social and material deprivation. The indicators included in calculating the DI were:

- The proportion of the district population that were children < 5;
- The proportion of the district population that were black African;
- The proportion of household heads in the district that were female;
- The proportion of household heads in the district with no formal education;
- The proportion of working-age population within the district that were unemployed;
- The proportion of the district population living in a traditional dwelling, informal shack or tent;
- The proportion of the district population with no piped water in their house or land;
- The proportion of the district population that with a pit, bucket or no toilet;
- The proportion of the district population with no access to electricity, gas or solar power for lighting, heating or cooking;

Based on the DI the countries’ 52 districts were ranked into 5 socio-economic quintiles, with 10 or 11 districts (20% of all districts) in each quintile. Districts in quintile 1 were most deprived and those in quintile 5 least deprived. The figure on the 3^rd^ page of this appendix shows the deprivation indices for all districts in South Africa, ranked into socio-economic quintiles.

The 2 decentralised sites included in this study were in Umzinyathi and Zululand districts. (Decentralised hospital 1 is in Umzinyathi and Decentralised 2 in Zululand districts respectively). As can be seen in Figure 1 on page 3 of this document these 2 districts were in the most deprived socio-economic quintile (SEQ 1) with deprivation indices of 4.6 and 4.2 respectively. Patients in the community-based mobile model of treatment were all in Umzinyathi district (DI = 4.6). Patients in the community-based clinic model were from all 4 districts where there was a decentralised hospital. So, in addition to the 2 districts already mentioned, this included Ugu (DI=3.8) and Umkhanyakude (DI=4.5), also were also in the lowest SEQ in the country.

Although the DI is based on data from 3 years before our study, the situation in these districts has not changed. In 2011, the South African Government’s National Development Plan (NDP) confirmed that the 4 districts with decentralised sites were amongst the poorest in the country.^2^ These districts with the poorest basic services were identified as priority districts for government and donor funding and activities. The 4 districts included in our study were amongst these 21 priority districts. Although the list was revised in 2014 the 4 study districts are still considered priority districts due to the poor level of services in these districts.

Although the Centralised Hospital is not in a district in SEQ1 and the district where it is based (eThekweni metro ) had a DI of 1.8 the year before the study commenced, patients in this hospital come from all over the province. Of the 7 districts referring to the centralised hospital 2 were in SEQ1 (Sisonke DI=4.1; Uthukela DI=3.7) and 2 were in SEQ2 (iLembe DI=3.6; Uthungula DI=3.5). Moreover, all the provinces districts except the metro were identified as priority districts in the NDP in 2011 and in the revised list in 2014.^2^

Having established that the study districts were in similar SEQs we felt confident in assuming that the socio-economic baseline characteristics of patients in the study were similar. We then examined a number of health indicators to determine whether the health services and the health of the district populations were similar. To do this we looked at the following health indicators during the course of the study:

**Per capita expenditure on primary health care (non-hospital):** We used this indicator to assess the extent of equity in the distribution of primary health care resources across districts.

**Baby PCR coverage:** This indicator measures the number of babies who are PCR tested 6 weeks after birth as a proportion of live births to HIV-positive women.

**Diarrhoea incidence in children <5 years:** We measured this incidence as diarrhoeal disease is one of the major killers of children in developing countries.

**HIV prevalence amongst antenatal clients tested:** This data provides information on HIV prevalence at a district level.

As can be seen in the table below the health service and health status indicators for the study districts were similar, suggesting that the health service and health status of the populations in these districts were similar. Furthermore, the variation in these indicators across all districts in the province was limited, suggesting that the patients referred to the centralised hospital came from districts with a similar health service and health status to the study districts. (The variation across the KwaZulu-Natal districts was far narrower than that across the districts of the rest of the country.)

**Table D: A comparison of health indicators across KwaZulu-Natal districts (2007-2011)**

| **District number** | **District name** | **PHC expenditure per capita**  **(in local currency - rands)** | | **Diarrhoea incidence <5 per 1000 children** | | **Early infant HIV diagnosis coverage** | | **% ANC clients tested positive for HIV** | |
| --- | --- | --- | --- | --- | --- | --- | --- | --- | --- |
|  |  | 2007/2008^3^ | 2010/2011^1^ | 2007/2008^3^ | 2010/2011^1^ | 2007/2008^3^ | 2010/2011^1^ | 2007/2008^3^ | 2010/2011^1^ |
| DC21* | Ugu | R275 | R410 | 370 | 98 | Not available for KZN | 45% | 37% | 25% |
| DC22 | uMgungundlovu | R280 | R465 | 702 | 171 |  | 47% | 41% | 30% |
| DC23 | Uthukela | R280 | R324 | 371 | 105 |  | 29% | 36% | 25% |
| DC24 | Umzinyathi | R265 | R395 | 472 | 109 |  | 35% | 32% | 22% |
| DC25 | Amajuba | R215 | R395 | 268 | 136 |  | 53% | 39% | 30% |
| DC26 | Zululand | R283 | R400 | 461 | 155 |  | 46% | 35% | 24% |
| DC27 | Umkhanyakude | R340 | R440 | 413 | 130 |  | 26% | 39% | 22% |
| DC28 | Uthungulu | R280 | R380 | 467 | 178 |  | 52% | 36% | 24% |
| DC29 | iLembe | R304 | R425 | 465 | 180 |  | 56% | 42% | 24% |
| DC43 | Sisonke | R420 | R405 | 270 | 130 |  | 26% | 34% | 22% |
| Metro | eThekweni | R360 | R500 | 371 | 190 |  | 41% | 42% | 31% |
|  | KZN range | R215-R360 | R324-R500 | 268-472 | 98-190 |  | 26-56% | 34-42% | 22-31% |
|  | Range in SA | R191-R636 | R324-R1095 | 5-702 | 25.4-256.3 |  | 0-100% | 7-42% | 6-36% |
|  | SA average | R300 | R514 | 254 | 109 | 26% | 52% | 24% | 22% |

* The shaded rows are those rows in which the decentralised hospitals were based.

KZN: KwaZulu-Natal

SA: South Africa

**REFERENCES**

1. Day C, Barron P, Massyn N, Padarath A, English R, editors. District Health Barometer 2010/11. Durban: Health Systems Trust; 2012.

2. National Planning Commission. National Development Plan: Vision for 2030. Pretoria: South African National Government; 2011. <http://www.npconline.co.za/medialib/downloads/home/NPC%20National%20Development%20Plan%20> (accessed 14 March 2016)

3. Day C, Barron P, Monticelli F, Sello E. The District Health Barometer 2007/08. Durban: Health Systems Trust; June 2009.

4. Laserson K, Thorpe L, Leimane V, et al. Speaking the same language: treatment outcome definitions for multidrug-resistant tuberculosis. Int J Tuberc Lung Dis 2005;9:640–5.

5. World Health Organisation. Guidelines for the programmatic management of drug-resistant tuberculosis. Emergency Update 2008. WHO/HTM/TB/2008.402. Geneva: World Health Organisation; 2008.
