# Supplementary material for: MDR-TB patients in KwaZulu-Natal, South Africa: Cost-effectiveness of 5 models of care
Source: PLoS One. 2018 Apr 18;13(4):e0196003. doi: 10.1371/journal.pone.0196003 (PMC5906004; doi:10.1371/journal.pone.0196003)
Supplement: S2 Appendix — (DOCX) [file pone.0196003.s005.docx]

**S2 Appendix: Additional methodology and regards regarding the propensity weighting**

Propensity scores were calculated separately for each pair of comparisons, that is: Decentralised 2 vs Centralised, Clinic vs Decentralised 2, Mobile vs Clinic, Decentralised 1 vs Mobile using logistic regression. All baseline variables presented in Table 2 were considered as potential variables which may explain the propensity of an individual to be in one treatment model vs another. In all four comparisons the variables included were previous TB treatment, HIV and ART status, baseline weight and smear positive microscopy at baseline. A fundamental assumption of propensity score analysis is that each individual should have a positive propensity of being allocated to a treatment model. Due to this, in any comparisons involving decentralised 1, clinic or mobile, all HIV-positive patients not on ART were subsequently omitted from the comparison group as well. Therefore, the only possible comparison which included HIV-positive individuals not on ART was that of Decentralised 2 compared to Centralised.

Standardised differences expressed as the percentage of bias between covariates in treatment groups for the unweighted cohort and the propensity score weighted cohort were compared to assess the success of the matching process. In the subsections below we present additional information regarding the number of patients included and the success of the propensity score weighting in removing baseline imbalances.

**Decentralised 2 vs Centralised**

249 patients in Decentralised 2 and the Centralised model had missing information for at least one of the following variables: weight, smear microscopy status, previous TB, HIV or ART status and were therefore omitted from the propensity score analysis. The remaining 474 patients from the centralised model and 120 patients from the decentralised 2 model were included in the propensity analysis. The comparison of the standardized differences between the variables of interest in Decentralised 2 and the Centralised model are presented for the original and propensity score weighted sample below. The two largest sources of imbalance were the proportion experiencing previous TB and smear positivity at baseline. In the weighted analysis, these differences were corrected for, resulting in biases less than 10% of all variables of interest. The overidentification test for overall balance in all covariates, indicated balance between the centralised and decentralised 2 model (p-value 0.938)

**Supplementary Table E: A comparison of the standardized differences between the variables of interest in** **Decentralised 2 and the Centralised model**

| Variables | Standardized differences between variables in Decentralised 2 and Centralised | |
| --- | --- | --- |
|  | Original | Weighted |
| HIV-positive, no ART | 0.039 | 0.033 |
| HIV-positive, on ART | 0.203 | - 0.059 |
| Weight | -0.168 | - 0.036 |
| Previous TB | -1.432 | 0.003 |
| Smear-positive microscopy | 0.412 | 0.030 |

**Clinic vs Decentralised 2**

142 patients in Decentralised 2 and Clinic models had missing information for at least one of the following: weight, smear microscopy status, previous TB, HIV or ART status and were consequently omitted from the propensity score analysis, resulting in a total of 24 patients from the clinic and 120 patients from decentralised 2. The largest sources of imbalance between these models of care were baseline weight and previous TB. The comparison of standardized differences between the variables of interest in the clinic and decentralised 2 models, are presented for the original and propensity score weighted cohort in Table 2 below, where all differences post weighting were less than 12%. The overidentification test for overall balance in all covariates, indicated balance between Decentralised 2 and the Clinic model (p-value 0.638)

**Supplementary Table F: A comparison of the standardized differences between the variables of interest in the Clinic and Decentralised 2 models**

| Variables | Standardized differences between variables in Clinic and Decentralised 2 | |
| --- | --- | --- |
|  | Original | Weighted |
| HIV-positive, on ART | - 0.320 | - 0.005 |
| Weight | - 0.415 | - 0.119 |
| Previous TB | 0.614 | 0.118 |
| Smear-positive microscopy | - 0.117 | 0.107 |

**Mobile vs Clinic**

One patient in the clinic had missing information for one of the co-variates and was omitted from the propensity score analysis, resulting in a total of 69 patients being included in the analysis (45 from the mobile and 24 from the clinic). The comparison of standardized differences between the variables of interest in the mobile and clinic models, are presented for the original and weighted cohort in Table 3B below. All differences post matching were less than 8%. The overidentification test for overall balance in all covariates, indicated balance between the clinic and mobile model (p-value 0.636)

**Supplementary Table G: A comparison of the standardized differences between the variables of interest in the Mobile and Clinic models**

| Variable | Standardized differences between variable in Mobile and Clinic | |
| --- | --- | --- |
|  | Original | Weighted |
|  |  |  |
| HIV-positive, on ART | - 0.091 | - 0.018 |
| Weight | 0.409 | 0.078 |
| Previous TB | - 0.271 | - 0.039 |
| Smear-positive microscopy | - 0.226 | - 0.072 |

**Decentralised 1 vs mobile**

Two patients in the decentralised 1 site had missing covariate information and 3 patients were HIV-positive and not on ART were omitted from the propensity score analysis, resulting in a total of 165 patients being studied The comparison of standardized differences between the variables of interest in decentralised 1 and the mobile models, are presented for the original and weighted cohort in Table 4B below. All differences post matching were less than 2,5%. The overidentification test for overall balance in all covariates, indicated balance between the mobile and decentralised 1 models (p-value 0.816)

**Supplementary Table H: A comparison of the standardized differences between the variables of interest in the Decentralised 1 and the Mobile model**

| Variables | Standardized differences between variable in Decentralised 1 and Mobile | |
| --- | --- | --- |
|  | Original | Weighted |
| HIV-positive, on ART | 0.315 | 0.004 |
| Weight | - 0.195 | 0.007 |
| Previous TB | 0.236 | - 0.009 |
| Smear-positive microscopy | 0.158 | - 0.023 |
